# Supplementary material for: Netcombin: An algorithm for constructing optimal phylogenetic network from rooted triplets
Source: PLoS One. 2020 Sep 18;15(9):e0227842. doi: 10.1371/journal.pone.0227842 (PMC7500971; doi:10.1371/journal.pone.0227842)
Supplement: S4 File — (ZIP) [file pone.0227842.s004.zip › SequenceData-Triplet/sequenceData-10-20-30-0-Triplet/length 300, numseq 30, rate 10^-7/6 seq.pdf]

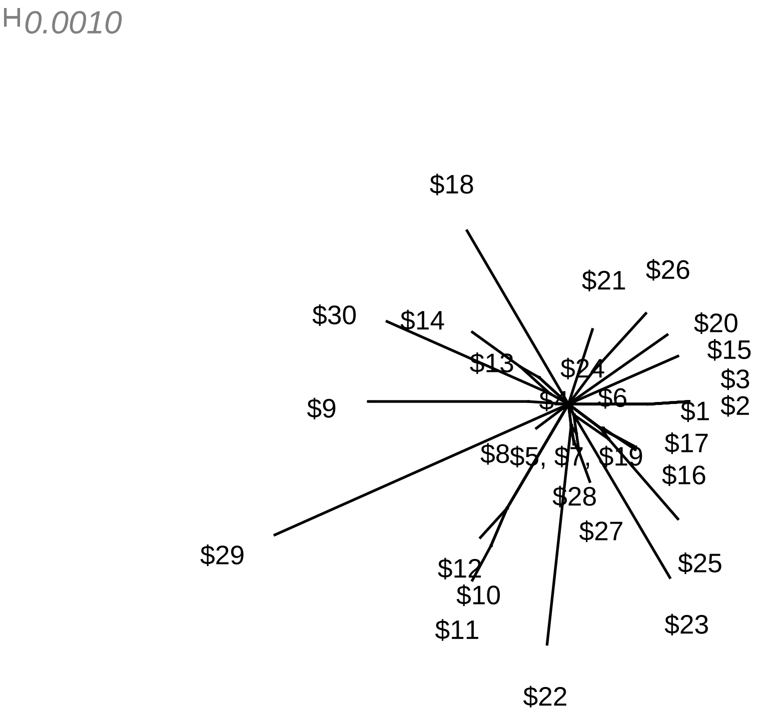
0.0010
$18
$21
$26
$30
$14
$20
$15
$13
$24
$3
$9
$4
$6
$1
$2
$17
$8
$5, $7, $19
$16
$28
$27
$29
$25
$12
$10
$11
$23
$22
